# Traumatized Syrian Refugees with Ambiguous Loss: Predictors of Mental Distress

**DOI:** 10.3390/ijerph18083865

**Published:** 2021-04-07

**Authors:** Anna Renner, David Jäckle, Michaela Nagl, Anna Plexnies, Susanne Röhr, Margrit Löbner, Thomas Grochtdreis, Judith Dams, Hans-Helmut König, Steffi Riedel-Heller, Anette Kersting

**Affiliations:** 1 Department of Psychosomatic Medicine and Psychotherapy, University of Leipzig, 04103 Leipzig, Germany; david.jaeckle@medizin.uni-leipzig.de (D.J.); michaela.nagl@medizin.uni-leipzig.de (M.N.); a.plexnies@gmail.com (A.P.); anette.kersting@medizin.uni-leipzig.de (A.K.); 2 Institute of Social Medicine, Occupational Health and Public Health (ISAP), Medical Faculty, University of Leipzig, 04103 Leipzig, Germany; susanne.roehr@medizin.uni-leipzig.de (S.R.); Margrit.Loebner@medizin.uni-leipzig.de (M.L.); Steffi.Riedel-Heller@medizin.uni-leipzig.de (S.R.-H.); 3 Department of Health Economics and Health Services Research, University Medical Center Hamburg-Eppendorf, 20246 Hamburg, Germany; t.grochtdreis@uke.de (T.G.); j.dams@uke.de (J.D.); h.koenig@uke.de (H.-H.K.)

**Keywords:** ambiguous loss, loss, refugees, boundary ambiguity, prolonged grief, PTSD, depression, anxiety, somatization

## Abstract

Refugees from war zones often have missing significant others. A loss without confirmation is described as an ambiguous loss. This physical absence with simultaneous mental persistence can be accompanied by economic, social or legal problems, boundary ambiguity (i.e., uncertainty about who belongs to the family system), and can have a negative impact on mental health. The aim of this study was to identify sociodemographic and loss-related predictors for prolonged grief, anxiety, depression, post-traumatic stress disorder (PTSD) and somatization in treatment-seeking Syrian refugees with post-traumatic stress symptoms in Germany experiencing ambiguous loss. For the present study, data were based on the treatment-seeking baseline sample of the “Sanadak” randomized-controlled trial, analyzing a subsample of 47 Syrian refugees with post-traumatic stress symptoms in Germany experiencing ambiguous loss. Sociodemographic and loss-related questions were applied, along with standardized instruments for symptoms of prolonged grief (ICG), anxiety (GAD-7), depression (PHQ-9), PTSD (PDS-5) and somatization (PHQ-15). Linear regression models were used to predict mental health outcomes. Having lost a close family member and higher boundary ambiguity showed a statistically significant association with higher severity in prolonged grief. The overall model for somatization reached statistical significance, while no predictor independently did. Boundary ambiguity showed a statistically significant positive association with depression, while the overall model showed no statistically significant associations. Boundary ambiguity and missing family members seemed to be important predictors for prolonged grief. These findings support the importance of reunification programs and suggest an inclusion of the topic into psychosocial support structures, e.g., including psychoeducational elements on boundary ambiguity in support groups for traumatized individuals and families experiencing ambiguous loss. Further research is needed for a more detailed understanding of the impact of ambiguous loss on refugee populations.

## 1. Introduction

By 2019, 6.6 million people had fled Syria due to the crisis that has lasted for nine years and that is still continuing [1]. Refugees are often affected by loss of status, income, home and social network as well as interpersonal losses [2,3]. In a population-based sample of Syrian refugees resettled in Sweden, the loss or disappearance of family members or loved ones was reported by 64%, forced separation from family or close friends were reported by 68% [3]. There are representative numbers showing that family separation is common among refugees in Germany [4]. A link between separation from marital partners and lower quality of life is reported in a study of Syrian refugees in Germany [5]. In addition, some affected persons describe the separation from the family as “traumatizing” [6]. However, to our knowledge, there are no reliable figures on how many refugees in Germany have missing relatives. The International Organization for Migration’s Missing Migrants Project recorded more than 30,000 border deaths globally, whilst the numbers of disappeared or missing migrants and refugees reach into the thousands. This however only includes fatalities at international borders and explicitly excludes a number of instances (such as deaths and disappearances) that occurred in the country of origin, after deportation or within refugee camps. Thus, the numbers cannot serve as overall information on how many migrants and refugees have disappeared [7].

Ambiguous loss (AL) is a sparsely researched topic in the field of refugees. It describes the phenomenon of loss without confirmation and thus potentially without closure [8,9,10]. The definition of AL is often broad, from incidents ranging from diseases like Alzheimer’s or addiction (physical presence with psychological absence) to disappearances during war and terrorism, incarceration, vanishing at sea or migration (physical absence with psychological presence [10,11]). Additionally, separation from family can be understood as AL when those persons are at permanent risk, e.g., when family members remain in Syria or are separated during the flight [12,13]. Utržan and Northwood [14] discussed the asylum process itself as a form of AL, since family members are physically absent and also psychologically not available due to uncertainty, fear and often guilt at not being able to support family members in severe situations. In our paper, we define ambiguous loss as the physical disappearance of a significant other during war, flight or resettlement without knowing whether the person is still alive. We focus on AL experienced by Syrian refugees based in Germany, since refugees from war zones often have missing family members or friends without having confirmation of their death [15,16].

As a consequence of AL, the lack of confirmation of death is often accompanied by the absence of rituals that may be important for the grieving process (e.g., funeral) [17]. Economic, social or legal problems can correlate with the missing of family members who have provided for family income [18]. Avoidant behavior may be encouraged in families and individuals due to fear of discovering the truth about the whereabouts of their loved ones [19]. On an emotional level, AL can be accompanied by guilt and helplessness [20]. Furthermore, the lack of clarity about the whereabouts of family members may complicate emotional or practical decisions that need to be taken for the future. This goes along with the phenomenon of not knowing who is in or out of a certain system (e.g., core family), which is described as boundary ambiguity [9]. Boundary ambiguity can be a consequence of AL, is stated to confuse family roles, determine the family’s level of stress and was associated with depression and anxiety earlier [21,22,23,24].

Research on the link between AL and mental health is still limited. AL was associated with prolonged grief, anxiety, depression, post-traumatic stress disorder (PTSD), substance abuse and stress-related illnesses and hence was considered a risk factor for mental health [8,9,10,12,20,25]. Boss [10] distinguished the experience of AL from PTSD in the sense that the trauma of uncertainty about loss persists. A systematic review by Lenferink, Keijser, Wessel, Vries and Boelen [26] that investigated disappearances due to war or state terrorism reported widely varying prevalence rates in a small number of studies assessing PTSD (1–67%), depression (3–88%), anxiety (1–65%), prolonged grief (7–23%) and somatic complaints (43%) in persons experiencing AL. Moreover, the authors did not find a statistically significant difference regarding psychopathology between relatives of disappeared persons and relatives of homicide victims. In contrast, Isuru et al. [12] found higher prevalence rates of depression and prolonged grief in persons who did not receive the mortal remains of the disappeared person in comparison to those who did after a tsunami in Sri Lanka. A study with relatives of persons who disappeared or were killed during the war in Bosnia–Herzegovina showed higher depression and traumatic grief scores in relatives of disappeared persons [19]. Furthermore, forced separation, loss or disappearance of family members or loved ones and the lack of current information about family members were predictors for mental health in refugees in Europe [3,27]. Other studies highlight that the disappearance of a significant other does not go hand in hand with psychopathology and that there are different ways of coping. For example, a study with relatives of disappeared persons in the Netherlands showed that acceptance of the disappearance, mental disengagement, emotional social support and venting emotions with others were helpful coping strategies [28]. 

Previous studies found mixed results regarding predictors for mental distress in persons with AL. Individual studies found female sex and higher age in persons experiencing AL to predict anxiety and depression but not prolonged grief or PTSD [12,29,30,31]. The relation to the missing person is one of the better-evidenced predictors of mental distress in relatives of disappeared persons. Thereby, spouses and parents seemed to be most affected by AL [12,25,26,29,32]. In addition, mental distress seemed to be related to a higher exposure to traumatic events (TE) [19,33]. On an individual level, emotion-focused coping strategies were associated with higher levels of anxiety, depression and stress in persons with AL compared to problem-focused strategies [27]. Regarding family strategies after AL, cooperative problem solving and being in control of dealing with adverse life events were associated with fewer avoidant PTSD symptoms and lower prolonged grief [29]. Ambivalence regarding whether the disappeared person died and a higher extent of hope that the person was alive showed higher prolonged grief [12,31]. Concurrently, Wayland, Maple, McKay, and Glassock [34] postulated hope as useful for dealing with AL. Lenferink et al. [26] formulated the hypothesis that violent disappearances might be associated with higher levels of grief compared to non-violent disappearances. 

The field of AL is under-researched, studies have shown contradictory findings and there is a call for more insights into correlates of psychopathology in relatives of disappeared persons [10,26,31]. To our knowledge, though refugees are often exposed to AL, there are hardly any studies addressing AL in refugee populations. There is a need to identify important factors that influence mental health and to derive clinical implications to enhance well-being of those affected. Hence, the aim of this study was to identify predictors for prolonged grief, anxiety, depression, PTSD and somatization in Syrian refugees with post-traumatic stress symptoms (PTSS) in Germany who experienced AL. 

## 2. Methods

### 2.1. Study Sample and Data Collection

Baseline data (*N* = 133) of the “Sanadak” randomized controlled trial, a project to evaluate the effectiveness of a self-help app for traumatized Syrian refugees, was used. The project was approved by the ethics committee of the Medical faculty of the University of Leipzig (reference number: 111/17-ek). Our sample consisted of the participants who answered “Yes” to the question “Is there a close person in your life (for example a relative, a partner or a friend) who is missing?”, resulting in a total sample size of *N* = 47. The sample was recruited using a multi-strategic approach and consisted of Syrian refugees living in the urban area of Leipzig, Halle/Saale and Dresden in Germany. Inclusion criteria were: Syrian refugee (18–65 years), experience of at least one TE, mild to moderate PTSS (PDS-5 = 11–59) and possessing a device compatible of running the app applied in the project. The exclusion criteria were: severe post-traumatic stress symptomatology (PDS-5 ≥ 60), severe depression (PHQ-9 ≥ 20), acute suicidality (Depressive Symptom Inventory-Suicidality Subscale/DSI-SS ≥ 3; [35]), current psychotherapy, psychiatric or psychopharmaceutical treatment and pregnancy. Trained Arabic native speakers collected the data in the form of an interview (11/2018–12/2019). Further information on the original study can be found elsewhere [36,37].

### 2.2. Instruments

A questionnaire was applied, including sociodemographic and loss-related questions as well as standardized instruments to assess the main outcomes prolonged grief, anxiety, depression, PTSD and somatization. The questionnaire was translated into Arabic and pretested based on the TRAPD model (translation, review, adjudication, pretesting, and documentation), except for the instruments PHQ-9, PHQ-15 and GAD-7, which were already available in Arabic [38]. Additionally, we assessed the total variability of TE by forming a sum score of the various categories mentioned in the PDS-5 [39]. Since different types of traumatization rather than frequency of events were assessed, we use the term “variability of TE” instead of the common expression “sum/number of TE”. Participants were asked about their age, sex, education (school years, highest school leaving certificate), family status, housing situation, residential and occupational status. Loss- and AL-related variables were assessed: number of missing persons, age of participant at the time of disappearance, age of the missing person, time passed since going missing, perceived closeness to the missing person (“not at all”—“very close”) and relation to the missing person (e.g., “partner”, “parent”, “close friend”). Further, participants were asked if they additionally lost one or more significant others. If respondents stated that they had more than one missing significant other, they were told to focus on the person whose disappearance has affected them the most.

Complicated grief was measured using the ICG (Inventory of Complicated Grief; Prigerson et al., 1995). This inventory consists of 22 items, each scoring on a 5-point Likert scale from 0 (never) to 4 (always), which are then summed up to a total score of max. 88 points, with a higher score indicating a higher symptom burden. We used a cut-off of >25 points as suggested by Prigerson et al., (1995) [40] to indicate higher impairment in social, mental, general and physical health functioning and bodily pain. Analogous to Lenferink et al. (2017) [41], we have adapted the items of the ICG to the disappearance instead of the death of a person. For example, item 3 was adapted from “I feel I cannot accept the death” to “I feel I cannot accept the disappearance”. Internal consistency (Cronbach’s α) in the present study for this instrument was 0.935.

As a measure for anxiety we used the GAD-7 (Generalized Anxiety Disorder Screener; Spitzer et al., 2006 [42]). This 7-item instrument measures anxiety symptoms using a 4-point Likert scale from 0 (not at all) to 3 (nearly every day) per item, with a maximum score of 21 points. A higher score indicates higher symptom burden, while a cutoff of ≥10 marks a probable anxiety diagnosis (Kroenke et al., 2010). Internal consistency (Cronbach’s α) in the present study was 0.870.

Depressive symptoms were assessed using the PHQ-9 (Patient Health Questionnaire 9; Kroenke et al., 2001 [43]), a 9-item questionnaire using a 4-point Likert scale from 0 (not at all) to 3 (nearly every day) with a maximum of 27 points. A higher score indicates higher symptom burden, while a cutoff of ≥10 indicates a probable depression diagnosis (Kroenke et al., 2010). Internal consistency (Cronbach’s α) in the present study was 0.827.

We assessed post-traumatic stress disorder using the PDS-5 (Post-Traumatic Diagnostic Scale-5; Foa et al., 1997 [39]). This instrument consists of 20 items assessing post-traumatic stress symptoms on a 5-point Likert scale from 0 (not at all) to 4 (6 or more times a week/severe) with a maximum score of 80, with a higher score indicating a higher symptom burden. A cutoff of ≥28 marks a probable PTSD diagnosis (Foa et al., 2016). Internal consistency (Cronbach’s α) in the present study was 0.868.

Symptoms of somatization were assessed using the PHQ-15 (Patient Health Questionnaire-15; Kroenke et al., 2002 [44]). This instrument originally consists of 15 items using a 3-point Likert scale from 0 (not at all) to 2 (more than half the days or nearly every day) with a total score of 30 points. Based on Beutel et al., (2019), the item ‘Menstrual cramps or their problems with your periods’ was removed to avoid gender bias in the outcome, leading to a total score of 28 instead of 30. A higher score represents higher symptom burden and a cutoff of ≥10 indicates a probable diagnosis of somatization (Kroenke et al., 2010). Internal consistency (Cronbach’s α) in the present study was 0.805.

As measure for boundary ambiguity we used a shortened, adapted and translated version of the “Boundary Ambiguity Scale for Wives of Men Declared Missing-in-Action (MIA)” [21], referred to as BAS (Boundary Ambiguity Scale) in the present study. The original version consists of 18 items covering changes in family life after the missing of a husband after a military mission. In our adapted version, the term “husband” was replaced by the term “close person”. The items were reduced to 11, excluding those not matching our target population, e.g., “I hope to remarry”. For our adapted version of this instrument, internal consistency (Cronbach’s α) was questionable with α = 0.643. In order to improve reliability, we analyzed average inter-item correlations in a first step, removing every item scoring less than 0.1. In accordance with Clark and Watson [45], we kept the remaining 7 items that were close to the mean value of 0.198, resulting in a Cronbach’s α of 0.750. The adapted version consisted of the items “I keep wondering if he/she could still be alive”, “I still believe that he/she is alive”, “I have always remained hopeful that he/she might return”, “I have felt it would be difficult, if not impossible, for me to find a new life without him/her”, “I think about him/her often”, “I will not be able to have closure until I know what happened to him/her” and “I have come to terms with the loss”. Statements were rated on a 5-point Likert scale from 0 (doesn’t apply at all) to 4 (fully applies) and then summed up to a total score (0–35). 

### 2.3. Statistical Analysis

For all statistical analyses, we used IBM-SPSS 25.0 statistical package for Windows (IBM, 2017). The following descriptive analyses were carried out for sociodemographics and all other variables: means, standard deviations, ranges and percent values. All available loss-related variables were included in the analysis as potential predictors. In a second step, Pearson’s correlation coefficients were calculated and variables that showed a statistically significant association with at least one of the main outcomes were included for all outcomes, as we aimed to have a consistent predictor selection (see Table 1). Hierarchical multiple regression analyses were conducted to examine predictors using the enter method. In a first step, sociodemographic variables (age, sex, education) were included as control variables, followed by loss-related predictors in a second step (relation to missing person, closeness to missing person, boundary ambiguity). After checking for regression assumptions, we found a violation of homoscedasticity. Therefore, the standard deviations and the 95% confidence intervals (95% CIs) were calculated using bootstrapping performed with 2000 iterations for all main outcomes. SPSS MT (Mersenne Twister) was applied to be able to replicate the calculations. The level of significance was set at *p* < 0.05. Dummy variables were coded for the variables age (young: 18–39 years; middle: 30–39; old: ≥40 years) and relation to the missing person (close family: partner, sibling, parent; other family: grandparents, parent-in-law, others; friends).

## 3. Results

### 3.1. Sample Characteristics

Table 2 and Table 3 give an overview of sociodemographic and loss-related data. Most participants were male (*n* = 31; 66.0%), with a mean age of 34.79 years (*SD* = 12.32; range = 19–64) and an average education of 13.74 years (*SD* = 3.52; range = 7–21) including vocational training or university studies. Participants had been missing an average of 2.42 (SD = 2.22; range = 1–10) persons, stating that they were mostly other relatives (*n* = 18; 38.3%) or close friends (*n* = 14; 29.8%). With 66.0% (*n* = 31), the majority of the sample knew or suspected reasons for the disappearance (e.g., imprisonment (by government), political activism, military service,). Of the participants, 27.7% (*n* = 13) stated that they did not know or suspect the reasons for the disappearance, 6.4% (*n* = 3) did not answer the question. While 59.6 % (*n* = 28) of the sample stated they knew or suspected which (groups of) people were involved in the disappearance, 34.0% (*n* = 16) did not know. Of those knowing or suspecting, the most frequently named groups were the Syrian government (*n* = 17) and terrorists/ISIS (*n* = 5), whereas, 6 persons gave no answers or answers that were unclear to the authors.

Detailed outcome categories for the measures ICG, GAD-7, PHQ-9, PDS-5, PHQ-15 and BAS can be seen in Table 4. The total score for ICG was M = 28.57 (*SD* = 17.90), with 42.6% (*n* = 20) scoring above the cutoff (>25). The total score of GAD-7 was M = 9.11 (*SD* = 5.36), with 40.4% (*n* = 19) people scoring above the cutoff (≥10). For PHQ-9, the total score was M = 10.13 (*SD* = 5.73), with 48.9% (*n* = 23) scoring above the cutoff (≥10). For PDS-5, the total score was M = 25.06 (*SD* = 12.68), with 34.0% (*n* = 16) scoring above the cutoff (≥28). Finally, for PHQ-15, the total score was M = 9.82 (*SD* = 5.27), with 55.3% (*n* = 26) scoring above cutoff (≥10). Correlations between the main measures ICG, GAD-7, PHQ-9, PDS-5, PHQ-15 as well as the predictors are displayed in Table 1.

### 3.2. Regression Models

Results of the bootstrapped hierarchical regression models for all outcomes are presented in Table 5. Having lost a close family member (β = 15.72, *p* < 0.05) and higher boundary ambiguity (β = 1.09, *p* < 0.01) were statistically significantly associated with higher severity in prolonged grief. The final model accounted for 59.3% (R^2^ = 0.59, *p* < 0.001) of the variance. None of the included variables statistically significantly predicted anxiety in the model. The final model for anxiety was not statistically significant (R^2^ = 0.17, *p* = 0.05). Boundary ambiguity showed a statistically significant positive association with depression (β = 0.35, *p* < 0.05), while the final model was not statistically significant (R^2^ = 0.24, *p* = 0.05). The predictors included did not show a statistically significant effect on PTSD. The final model for PTSD was statistically not significant (R^2^ = 0.20, *p* = 0.05). No factors included in the model predicted somatization independently, while the final model was statistically significant (R^2^ = 0.40, *p* < 0.05).

## 4. Discussion

The aim of this study was to identify predictors for prolonged grief, anxiety, depression, PTSD and somatization in Syrian refugees with post-traumatic stress symptoms in Germany experiencing AL. Our results indicate that boundary ambiguity and missing family members are predictors for prolonged grief. The overall model for somatization was statistically significant while no predictor independently reached statistical significance. Boundary ambiguity showed a statistically significant positive association with depression, while the overall model showed no statistically significant associations.

In attempting to interpret the results of the analyses, it is first necessary to point out some specific characteristics of the sample. On one hand, sample size was small and hence may have led to an underestimation of the impact of individual variables or the overall models. It is possible that small effects (e.g., on anxiety, depression, PTSD or somatization) could not show up. On the other hand, due to the need in the Sanadak trial to include only individuals with at least mild PTSS, we cannot make any statements about Syrian refugees without PTSS. The exclusion of persons with severe depression and PTSD may have led to an underestimation of the predictors’ impact on the outcomes. The very specific inclusion- and exclusion-criteria of the study make the predictive value of our findings high for the very specific group of Syrian refugees with PTSS experiencing AL, however the findings may not be generalizable to other refugee populations. Additionally, it should be noted that while boundary ambiguity has been the subject of multidisciplinary research for over 40 years, the definition of the concept has been very broad and varied. In contrast, the selection of valid measurement instruments is very limited. The most commonly used measurement instrument is the BAS, which has been adapted many times. In the literature there is a call for a standardized version with good psychometric properties [22]. In addition to these difficulties in capturing the concept of boundary ambiguity, no version is adapted to the realities of refugees’ lives after experiencing war and flight, so we had to adapt the instrument. A validation of the instrument would have been desirable, however, the study lacked adequate measures due to the fact that we used baseline data of a RCT that primarily aimed at evaluating the efficacy of a self-help app. Therefore, results must be interpreted with caution. 

To our knowledge, our study is the first to report boundary ambiguity as a relevant predictor for prolonged grief in Syrian refugees with PTSS in Germany. Heeke et al. [31] reported the degree of hope for survival of the missing person to be associated with prolonged grief but not with depression earlier. Isuru et al. [12] found not being sure whether a person was dead or alive and the belief that the missing person was still alive to predict higher prolonged grief. Since the extent of hope and the belief whether a person is still alive are both components of the concept of boundary ambiguity [21], our results support and connect these findings. However, Isuru et al. [12] also found an association between the belief of the status of the missing person and depression. Since the overall depression model in our study was not statistically significant, we could not replicate this result. 

Regarding the relationship to the missing person, missing persons being a close family member (i.e., partner, child, parent, sibling) as compared to other relatives (i.e., grandparent, parent-in-law, others) predicted higher prolonged grief in this study while the missing person being a friend did not. This is an important finding since most previous research was on family members only or did not distinguish between family and friends [12,25,26,28]. Furthermore, the finding points in the same direction as a study by Georgiadou et al. [5], which showed that family separation is associated with lower quality of life among Syrian refugees. Interestingly, the closeness felt to the missing person did not predict higher prolonged grief in our sample. One possible hypothesis could be that these findings illustrate that organizational and legal needs and problems, which are greater when a member of the nuclear family is missing, are more likely to contribute to psychological distress than emotional closeness to the missing person.

In contrast to previous research [12,29,30,31], we did not find female sex and older age to predict anxiety and depression. Analogous to these previous results, it was also no predicting factor for PTSD or prolonged grief in our study. In line with Heeke et al. [31], the educational level was unrelated to prolonged grief. It is noteworthy that we did not find sociodemographic factors as predictors for mental distress in our sample at all. Since the effect of sociodemographic factors on mental health (e.g., prolonged grief) is well studied [46], the question arises as to the discrepancy with our research. One explanation might once again be the small sample size so that minor differences could not show in the regression analysis. The small to mediate correlations between sociodemographic variables and mental health outcomes in the bivariate analysis support this hypothesis. Further, it is possible that men were more frequently involved in military operations in Syria and thus potentially traumatic events than women, whereby the higher burden on women caused by gender-related factors would no longer be visible due to a war-based higher burden on men. However, the reports on wartime burdens specific to women speak against this hypothesis. By analogy, it can be hypothesized that younger persons were more likely to have been actively involved in the war, thus leveling out increasing age as a risk factor. Another explanation could also be the specific help-seeking sample, as only individuals who were interested in an online mental health support intervention responded. One assumption would be that rather “young at heart” persons are interested in such an innovative form of intervention. Regarding socioeconomic status, it could be hypothesized that the high overall burden in the help-seeking sample levels out the protective function of a higher income.

### 4.1. Strengths and Limitations

This study takes an important step by examining the still largely unexplored field of AL in the under-researched group of refugees with PTSS. The highly specific target group of Syrian refugees in Germany with PTSS experiencing AL allows the derivation of concrete implications for supporting a vulnerable subgroup of the largest group of refugees in Germany since 2013 [46]. The study may also contribute to a better understanding of the role that boundary ambiguity plays in prolonged grief, which may provide valuable information for clinical practice. Additionally, we adapted the original version of the boundary ambiguity measure by Boss et al. [21] to better serve our target group.

As discussed above, the most relevant limitation of our study is the small sample size, which may have led to an underestimation of the impact of included variables and did not allow including further possibly important factors in the analyses. Already the existing number of predictors could be quite high for the regression models with our small sample size. In addition, the exclusion of severely depressed persons and the inclusion criterion of having experienced at least one traumatic event may have led to bias. Further sources of bias could be the cross-sectional nature of our data and the use of self-report questionnaires. The sample consists of help-seeking individuals and is not representative for the general population of refugees based in Germany; therefore, generalization of the results is not appropriate. Although our measure of boundary ambiguity was adapted to the target population, it still lacks proper evaluation of test criteria, was not validated for the target group and the adaption process could use expert-based refinement. Additionally we would like to point out that the cultural, political, and social context of the authors has most likely influenced the research question as well as the interpretation of the results.

### 4.2. Implications

Since having lost a family member and boundary ambiguity are risk factors for prolonged grief in this study, we suggest supporting the search for missing relatives at political and organizational levels, for example through tracing services and reunification programs (e.g., by the German Red Cross). Additionally, psychosocial support groups including psychoeducation on boundary ambiguity and mental health could help to establish and expand coping mechanisms for dealing with ambiguous loss and strengthen social support structures. A systemic approach for the whole family system dealing with AL could be helpful, since prolonged grief is also defined as a type of AL [11] and can hence have adverse effects on the mental health of other family members. 

### 4.3. Further Research

In our help-seeking sample of Syrian refugees with PTSS in Germany, 35.6% (*n* = 47) of a total of *N* = 133 persons experienced AL. When interpreting this data, it should be kept in mind, that the sample is not representative and may be affected by a help-seeking bias. Therefore, future studies with representative samples are necessary to shed more light on the prevalence of AL among Syrian refugees. Moreover, further research should be carried out with a greater sample size to also enable detection of smaller effects of loss-related variables on anxiety, depression, PTSD, and somatization. A comparison between refugees with and without symptoms of post-traumatic stress regarding their boundary ambiguity after AL would be interesting to better understand the association between boundary ambiguity and psychopathology and to make results more generalizable. Further research should also include more potential predictors for psychopathology such as the time since, or the type of disappearance, but also potential resources such as social support or religiosity. Given that the whereabouts of many family members of Syrian refugees is unclear, family separation and ambiguous loss are probably closely connected in this population. Family separation was described as important mental health stressor before [5,6], hence, an investigation of the relationship between family separation and boundary ambiguity could contribute to a better understanding of AL in refugee populations. A group comparison between refugees who have experienced confirmed loss and ambiguous loss would be a further step towards understanding the impact of AL on mental health. Additionally, validation of the adapted version of the boundary ambiguity measure would be desirable. 

## 5. Conclusions

The results of our study indicate that boundary ambiguity and the missing of a family member could be predictors for prolonged grief in Syrian refugees with PTSS in Germany. These findings support the importance of tracing services and reunification programs (e.g., by the German Red Cross) to help those affected to get clarity about the whereabouts of disappeared persons. The topic of AL should be included in psychosocial support structures, for example psychoeducation and thematic exchange in support groups. Systemic approaches that support families in clarifying their roles and finding functional coping styles to deal with the ambiguous loss could be particularly promising. Further research is needed for a more detailed understanding of the impact of AL on refugee populations.

## Figures and Tables

**Table 1:** Pearson correlations of predictors and mental health outcomes.

	Complicated Grief (ICG)	Anxiety (GAD-7)	Depression (PHQ-9)	PTSD (PDS-5)	Somatization (PHQ-15)
Age	−0.036	0.056	0.004	0.026	0.245
Sex (m/f)	−0.119	0.147	0.087	0.143	0.267
School education	−0.145	−0.164	−0.057	−0.195	−0.396 **
Relation to missing person (Family vs. other)	0.594 **	0.318 *	0.298 *	0.339 *	0.382 **
Relation to missing person (Friend vs. other)	−0.094	−0.152	−0.110	−0.074	−0.295 *
Closeness to missing person	0.313 *	0.100	0.042	−0.019	0.248
Boundary ambiguity (BAS)	0.598 **	0.300 *	0.435 **	0.322 *	0.396 **
Complicated Grief (ICG)	1	0.618 **	0.636 **	0.544 **	0.442 **
Anxiety (GAD-7)	0.618 **	1	0.841 **	0.771 **	0.579 **
Depression (PHQ-9)	0.636 **	0.841 **	1	0.808 **	0.558 **
PTSD (PDS-5)	0.544 *	0.771 **	0.808 **	1	0.675 *
Somatization (PHQ-15)	0.442 **	0.579 **	0.558 **	0.675 *	1

Note: *N* = 47 adult Syrian refugees in Germany experiencing AL; * *p* < 0.05; ** *p* < 0.01.

**Table 2:** Sociodemographic information.

Characteristics	*n* (%)
Age (categories)	
<30 years	23 (48.9)
≥30 years	24 (51.1)
Sex	
Male	31 (66.0)
Female	16 (34.0)
Highest education	
<12 years	18 (38.3)
≥12 years	29 (61.7)
Family status	
Single	23 (48.9)
Married	20 (42.6)
Divorced	1 (2.1)
Widowed	1 (2.1)
I don’t know	2 (4.3)
Housing situation	
Alone	12 (25.5)
With others	35 (74.5)
Residential status	
Tolerance of stay (Duldung)	0 (0.0)
In asylum procedure (Gestattung)	5 (10.6)
Residency permit (Erlaubnis)	37 (78.7)
Other	4 (8.5)
Employment	
Unemployed	30 (63.8)
Employed	15 (31.9)

Note. *N* = 47 adult Syrian refugees; *n* (*%*) may vary in total due to missing values in single items.

**Table 3:** Loss related information.

Characteristics	M/SD/Range	*n* (%)
Number of missing persons	2.42/2.22/1–10	
Age at the time of missing (years)	28.67/12.39/9–57	
Age of missing person (years)	32.41/12.33/3–64	
Time passed since missing (years)	5.25/2.87/0.08–14.33	
Perceived closeness to missing person		
Not at all		5 (10.6)
A little close		4 (8.5)
Rather close		3 (6.4)
Near		11 (23.4)
Very close		22 (46.8)
Relation to missing person ^a^		
Partner		2 (4.3)
Own child		2 (4.3)
Parent		2 (4.3)
Siblings		3 (6.4)
Mother-/father-in-law		1 (2.1)
Other relatives		18 (38.3)
Close friend		14 (29.8)
Other ^b^		3 (6.4)
Loss of a close person		
Yes		41 (87.2)
No		3 (6.4)
Number of persons lost	7.59/10.47/1–55	

Note. *N* = 47 adult Syrian refugees; *n* (%) may vary in total due to missing values in single items; ^a^ Grandparents are not listed, as none were reported missing; ^b^ “Other” includes all kinship relationships in addition to those listed in the tables.

**Table 4:** Prevalence rates of mental health burden.

Measures	M/SD/Range	*n* (%)
Boundary Ambiguity Scale (BAS)	12.90/6.40/1–27	-
Complicated Grief (ICG > 25)	28.57/17.90/1–61	
Yes		20 (42.6)
No		24 (51.1)
Anxiety (GAD-7 ≥ 10)	9.11/5.36/2–21	
Yes		19 (40.4)
No		28 (59.6)
Depression (PHQ-9 ≥ 10)	10.13/5.73/0–24 ^a^	
Yes		23 (48.9)
No		24 (51.1)
PTSD (PDS-5 ≥ 28)	25.06/12.68/6–55 ^a^	
Yes		16 (34.0)
No		31 (66.0)
Somatization (PHQ-15 ≥ 10)	9.82/5.27/1–23	
Yes		26 (55.3)
No		21 (44.7)

Note. *N* = 47 adult Syrian refugees; *n* (*%*) may vary in total due to missing values in single items. ^a^ Ranges in this sample differ from the values used as inclusion criteria in the screening sample (PDS min. 11; PHQ max. 20), as it relates to a later survey date (baseline).

**Table 5:** Results from bootstrapped multiple linear hierarchical regression analyses (Model 2).

Variable	ΔR^2^ Step 1	ΔR^2^ Step 2	Total R^2^ (Adjusted R^2^)	B	*SE*	95% BCaCI
Complicated Grief ^†^	0.212	0.381 ***	0.593 (0.512) ***			
Age, young (30–39 vs. 18–29)				10.47	5.91	−1.99, 24.01
Age, old (30–39 vs. ≥40)				12.46	6.40	1.42, 24.93
School education (0 = <12 y.; 1 = 12 y.)				−0.86	4.53	−10.32, 9.92
Sex (0 = male; 1 = female)						
Relation to missing person (0 = family; 1 = other)				15.72 *	6.02	2.30, 26.32
Closeness to missing person				2.34	1.27	−0.07, 5.45
Boundary ambiguity				1.09 **	0.33	0.45, 1.77
Anxiety	0.091	0.076	0.167 (0.005)			
Age, young (30–39 vs. 18–29)				1.58	2.14	−3.46, 5.94
Age, old (30–39 vs. ≥40)				2.01	2.64	−2.94, 7.04
School education (0 = <12 y.; 1 = 12 y.)				−2.10	2.08	−5.99, 1.66
Sex (0 = male; 1 = female)				0.52	2.04	−3.97, 4.21
Relation to missing person (0 = family; 1 = other)				1.72	2.47	−2.57, 6.00
Closeness to missing person				−0.15	0.68	−1.47, 0.95
Boundary ambiguity				0.18	0.16	−0.12, 0.59
Depression	0.080	0.162	0.242 (0.094)			
Age, young (30–39 vs. 18–29)				0.73	2.14	−2.27, 6.37
Age, old (30–39 vs. ≥40)				2.59	2.37	−1.51, 6.53
School education (0 = <12 y.; 1 = 12 y.)				−1.98	1.87	−5.69, 1.07
Sex (0 = male; 1 = female)				−0.28	2.07	−4.62, 3.72
Relation to missing person (0 = family; 1 = other)				0.73	2.74	−4.25, 5.40
Closeness to missing person				−0.38	0.72	−1.77, 0.80
Boundary ambiguity				0.35	0.14	0.43, 0.7
PTSD	0.060	0.144	204 (0.049)			
Age, young (30–39 vs. 18–29)				7.59	5.36	−9.99, 11.65
Age, old (30–39 vs. ≥40)				3.18	5.53	−7.37, 13.28
School education (0 = <12 y.; 1 = 12 y.)				−3.38	4.45	−13.02, 4.73
Sex (0 = male; 1 = female)				0.94	4.16	−7.03, 8.53
Relation to missing person (0 = family; 1 = other)				8.52	6.92	−5.54, 21.65
Closeness to missing person				−1.32	1.43	−4.05, 0.87
Boundary ambiguity				0.41	0.33	−0.21, 1.18
Somatization	0.238 *	0.161	0.399 (0.282)			
Age, young (30–39 vs. 18–29)				0.03	2.07	−4.25, 4.05
Age, old (30–39 vs. ≥40)				1.92	2.21	−2.21, 6.51
School education (0 = <12 y.; 1 = 12 y.)				−2.87	1.72	−6.41,−0.06
Sex (0 = male; 1 = female)				1.92	1.65	−1.48, 5.14
Relation to missing person (0 = family; 1 = other)				2.91	2.27	−1.44, 7.28
Closeness to missing person				0.30	0.58	−0.92, 1.25
Boundary ambiguity				0.23	0.13	−0.01, 0.57

Note: complicated grief (ICG), anxiety (GAD-7), depression (PHQ-9), PTSD (PDS-5), somatization (PHQ-15); *B* = unstandardized coefficient; *SE* = standard error; BCaCI = bias-corrected and accelerated 95% confidence interval; R^2^ (R square) = percentage of variance explained by the model; AdjR^2^ (adjusted R square) = adjusted for the number of terms included; ΔR^2^(Delta R Square) = change in R square; * *p* < 0.05. ** *p* < 0.01. *** *p* < 0.001; ^†^ *n* = 43 adult Syrian refugees; *n* = 44 adult Syrian refugees.

## Data Availability

The data presented in this study are available on request from the corresponding author. The data are not publicly available due to participant confidentiality and privacy.

## Abbreviations

AL	Ambiguous loss
PTSD	Post-Traumatic stress disorder
PTSS	Post-Traumatic stress symptoms
TE	Traumatic events
